# THE USE OF CREATIVITY WITH CLIENTS WITH DEMENTIA TO ENHANCE STUDENT LEARNING AND POSITIVE PERCEPTIONS

**DOI:** 10.1093/geroni/igz038.2661

**Published:** 2019-11-08

**Authors:** Megan Foti, Michelle Ghaul

**Affiliations:** 1 Stockton University, Galloway, New Jersey, United States; 2 Fox Rehabilitation, Galloway, New Jersey, United States

## Abstract

Funding and productivity demands combined with curative approaches of medicine can make working with older adults less appealing to health professionals (Samra et al, 2013 George et al, 2013). It is essential to recognize and address such perceptions to promote the provision of quality, humanistic healthcare. Educators can impact perceptions by facilitating innovative opportunities for interaction with older adults, especially those in dementia care. Creative therapies, such as art, music, and storytelling provide opportunities for reminiscence and self-expression and have been proven to yield potential psychosocial benefits for people with dementia including enhanced well-being, lessened cognitive decline, decreased anxiety and depression, and improvements in memory, social interaction, orientation, and cognitive functioning. (Phillips et al, 2010; Subramanium, Trentham, n.d.; Woods, & Whitaker, 2013). Similarly, these interventions yield benefits for facilitators such as increased comfort and higher levels of humanistic care for clients with dementia (George et al, 2013, & 2014). The proposed presentation will provide evidence related to the benefits of creative therapies for people with dementia and highlight methods for integrating this into healthcare curricula in addition to supporting evidence and positive outcomes.

